# Early creatinine dynamics for stratifying acute kidney injury trajectories in patients with systemic lupus erythematosus: an exploratory multicenter retrospective study

**DOI:** 10.3389/fimmu.2026.1910588

**Published:** 2026-08-17

**Authors:** Rui Yue, Huiting Yin, Xueqin Zhang, Chang Li, Deqian Meng, Ju Li, Donghui Zheng, Shanshan Liu, Kai Wang

**Affiliations:** 1 Department of Rheumatology and Immunology, The Affiliated Huaian No.1 People’s Hospital of Nanjing Medical University, Huaian, China; 2 Department of Rheumatology and Immunology, The Huaian Clinical College of Xuzhou Medical University, Huaian, China; 3 Department of Medical Laboratory, The Affiliated Huaian No.1 People’s Hospital of Nanjing Medical University, Huaian, China; 4 Department of Nephrology, The Affiliated Huaian No.1 People’s Hospital of Nanjing Medical University, Huaian, China

**Keywords:** acute kidney injury, exploratory external assessment, hydroxychloroquine, latent class mixed model, machine learning, systemic lupus erythematosus, trajectory risk stratification

## Abstract

**Objective:**

Patients with systemic lupus erythematosus (SLE) exhibit heterogeneous renal courses after acute kidney injury (AKI). This study applied latent class mixed modeling to identify AKI trajectory phenotypes in SLE, developed a risk-stratification model using early post-AKI data, and explored whether hydroxychloroquine (HCQ) use is associated with AKI trajectory class membership.

**Methods:**

This multicenter retrospective study used MIMIC-IV for model development and internal assessment and eICU, NWICU, and local cohorts for exploratory external assessment. Adult SLE patients with AKI were included. The primary model was a race-free, no-imbalance-correction LASSO logistic regression model. Performance was assessed with AUC, Brier score, calibration intercept, and calibration slope. Bootstrap optimism correction was applied. A non-overlap sensitivity analysis examined the influence of structural predictor-outcome overlap. HCQ was evaluated using multivariable logistic regression and propensity score matching.

**Results:**

Among 279 MIMIC patients, three AKI trajectories were identified (progressive 9.3%, recovery 19.4%, stable 71.3%). The primary race-free LASSO model achieved an apparent AUC of 0.761 (optimism-corrected: 0.706) and a Brier score of 0.086 (corrected: 0.092). When assessed against a non-overlapping post-48-hour renal endpoint, discrimination diminished substantially (AUC 0.593). Exploratory external assessments were event-limited: the main SLE external discrimination subsets contained 6 events in eICU, 3 events in NWICU, and 1 event in the local cohort; therefore, these estimates were considered descriptive rather than definitive validation. HCQ showed an exploratory association not interpretable as causal.

**Conclusion:**

This study provides an exploratory risk-stratification framework for early AKI trajectory patterns in SLE. The apparent discrimination was substantially attenuated after removing predictor-outcome overlap and adjusting for optimism. External assessments were event-limited and should be interpreted as exploratory transportability assessments rather than definitive evidence of generalizability.

## 1 Introduction

Systemic lupus erythematosus is a chronic autoimmune disease characterized by multisystem involvement, and renal involvement is one of the major determinants of long-term prognosis (1, 2). Lupus nephritis is associated with increased risks of chronic kidney disease, end-stage kidney disease, and mortality (2, 5). Beyond classical lupus nephritis, patients with SLE may also develop AKI during hospitalization or intensive care as a consequence of infection, hemodynamic instability, nephrotoxic drug exposure, disease activity, and complications related to immunosuppressive therapy (3, 4). Once AKI occurs, subsequent renal recovery is highly variable. Some patients recover rapidly, whereas others experience persistent deterioration or even require kidney replacement therapy. Therefore, early identification of patients with SLE-AKI who are at high risk for progressive renal dysfunction is clinically important.

Current AKI definitions and staging systems, including the KDIGO criteria, are based mainly on changes in serum creatinine or urine output within specific time windows (5, 6). Although these criteria are useful for identifying AKI occurrence and severity, they do not fully characterize the longitudinal evolution of renal function after AKI. In patients with SLE, disease activity, immune-mediated renal injury, infection-related inflammation, hemodynamic changes, medication exposure, and baseline renal reserve may interact in complex ways. As a result, serum creatinine patterns after AKI may show marked heterogeneity. Compared with single time-point assessments or static staging systems, trajectory-based risk assessment may therefore better reflect the clinical evolution of SLE-AKI (7).

Latent class mixed modeling can identify unobserved subgroups of patients who share similar longitudinal patterns (8). In recent years, machine learning methods have been increasingly applied to predict AKI onset, progression, and recovery (9–12). However, most existing models were not specifically developed for patients with SLE and seldom incorporate SLE-specific treatment factors. HCQ is a cornerstone therapy for SLE, and previous studies have shown that it can reduce disease activity, prevent flares, and improve long-term outcomes (13–16). Both the 2023 EULAR recommendations for SLE management and the 2024 KDIGO guideline for lupus nephritis recommend HCQ use in the absence of contraindications (17, 18). Nevertheless, the relationship between HCQ use and dynamic AKI trajectories in patients with SLE remains unclear.

In this study, we used the MIMIC-IV database to identify AKI trajectory phenotypes in patients with SLE-AKI, developed an interpretable early risk-stratification model for progressive trajectory membership, and performed exploratory external assessments in the eICU, NWICU, and local cohorts. We further examined the association between HCQ use and AKI progression and explored whether this association was specific to the SLE disease context.

## 2 Methods

### 2.1 Study design and data sources

This was a multicenter retrospective cohort study. The primary model development and internal assessment were based on the MIMIC-IV database. Exploratory external assessment was performed using the eICU Collaborative Research Database, the NWICU cohort, and a local hospital-based SLE-AKI cohort. Analyses of publicly available databases complied with the corresponding data use agreements, and the local data analysis was conducted in accordance with institutional ethical requirements. Details regarding data extraction, variable definitions, diagnostic coding, medication mapping, and reproducibility procedures are provided in the Supplementary Methods.

### 2.2 Study population

In MIMIC-IV, we included adult patients aged 18 years or older who had an ICD-coded diagnosis of SLE, developed AKI during hospitalization, and had sufficient serum creatinine measurements within 7 days after AKI onset. AKI was defined according to the KDIGO serum creatinine criteria, namely an increase in serum creatinine of at least 0.3 mg/dL within 48 hours or an increase to at least 1.5 times baseline within 7 days. Patients with chronic dialysis, a history of kidney transplantation, or insufficient key data were excluded. To compare model performance between SLE and non-SLE AKI populations and to explore whether HCQ-related associations differed according to disease context, a non-SLE AKI control cohort was also constructed.

### 2.3 Identification of AKI trajectories

AKI trajectories were identified using serial serum creatinine measurements obtained within 7 days after AKI onset. To reduce the effect of skewed distributions, creatinine values were log-transformed before being entered into the LCMM. Models with one to five latent classes were fitted, and the final number of classes was selected by jointly considering log-likelihood, Akaike information criterion (AIC), Bayesian information criterion (BIC), entropy, minimum class proportion, mean posterior classification probability, convergence status, and clinical interpretability. The resulting classes were named progressive, recovery, and stable according to the direction and shape of the creatinine trajectories. In external cohorts, trajectory labels were generated using either independent LCMM or rule-based labeling aligned with the MIMIC stable trajectory range, depending on data structure; the detailed procedures are described in the Supplementary Methods.

### 2.4 Risk-stratification target and candidate variables

The primary risk-stratification target was membership in the progressive AKI trajectory. Because the number of progressive events in the derivation cohort was limited, model development was framed as a binary classification task: progressive trajectory versus non-progressive trajectory. The non-progressive group included both recovery and stable trajectories. Candidate variables included demographics, comorbidities, medication exposures, vital signs, laboratory tests, and early post-AKI creatinine dynamics. The 48-hour creatinine slope was calculated by fitting a linear trend to available serum creatinine values obtained within 0–48 hours after AKI onset and was used to characterize the early rate of renal function change.

In the primary analysis, LASSO logistic regression without class imbalance correction was used for feature selection and model fitting. The candidate predictor pool included clinically available variables within 48 hours of AKI onset, limited by the number of progressive events (n=26). Race/ethnicity was excluded from the primary model to avoid using a social construct as a core predictor. The final primary model was a race-free LASSO logistic regression model. The final feature set was determined by integrating LASSO results with clinical interpretability, variable availability, and cross-database transportability. The revised primary model included five core variables: 48-hour creatinine slope, vasopressor use, history of hypertension, HCQ use, and vancomycin use. Race/ethnicity was excluded from the primary model and retained only in a secondary sensitivity analysis. Imputation, standardization, categorical encoding, and hyperparameter tuning were performed within the training or cross-validation pipeline to minimize information leakage. Class-imbalance procedures, when evaluated in sensitivity analyses, were also applied only within the training folds.

### 2.5 Model development and statistical analysis

Four modeling approaches were compared: LASSO logistic regression, standard logistic regression, random forest, and XGBoost. All models were trained using the same five core variables and generated a predicted probability of belonging to the progressive AKI trajectory. Internal performance was evaluated using repeated stratified cross-validation, with the area under the receiver operating characteristic curve (AUC) as the primary metric. Exploratory external assessment results were reported as AUC values and interpreted in conjunction with sample size, event count, and confusion matrices. A non-overlap sensitivity analysis was performed to evaluate the influence of structural predictor-outcome overlap. The primary model risk score was applied to predict a post-48-hour hard renal endpoint (creatinine doubling or death) that did not incorporate creatinine values from the predictor window. Discrimination and calibration were assessed.

**Figure 1:**
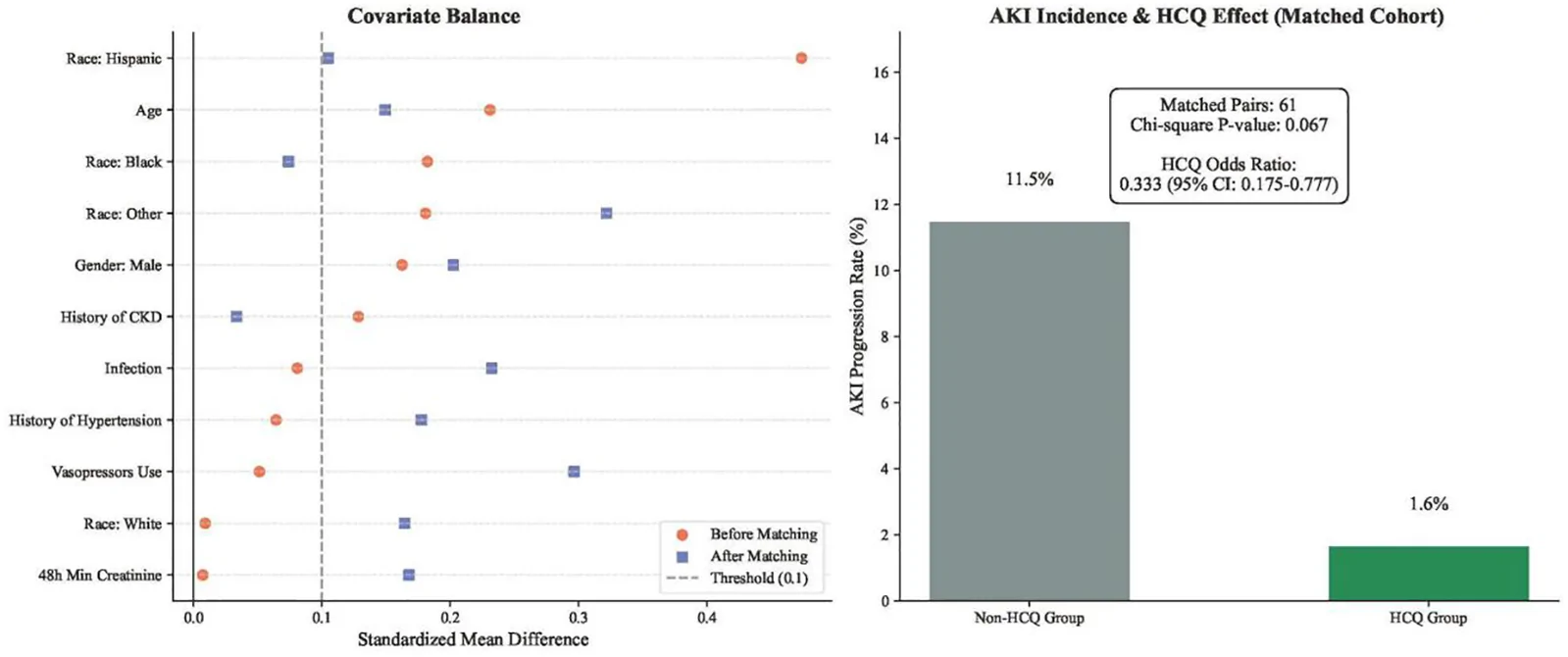
Propensity score matching analysis of HCQ use and AKI progression. The progression rate was 11.5% in the non-HCQ group and 1.6% in the HCQ group, corresponding to an exploratory OR of 0.333 (95% CI, 0.175–0.777) for HCQ use. This observational association should not be interpreted as causal.

To evaluate the independent associations between core variables and AKI progression, multivariable logistic regression was fitted in the MIMIC SLE cohort. Results were reported as odds ratios (ORs) and 95% confidence intervals (CIs). To further evaluate the observational association between HCQ use and AKI progression, propensity score matching was performed. In addition, an interaction model including SLE status, HCQ use, and the SLE × HCQ interaction term was constructed in the combined SLE and non-SLE cohort. The HCQ propensity score analysis is shown in **Figure 1**, and the interaction analysis is shown in **Supplementary Figure 3**. Details of propensity score matching, bootstrap procedures, sensitivity analyses, and exploratory external assessment are provided in the Supplementary Methods.

Given the limited number of progressive AKI events, the events-per-variable ratio was calculated for the revised primary SLE risk-stratification model. The MIMIC SLE-AKI cohort included 26 progressive events and the revised primary five-variable model included five core predictors, corresponding to an EPV of 5.2. For the previous six-variable race-including sensitivity model, the EPV was 4.3. To further assess potential overfitting, bootstrap optimism correction with 1000 resamples was performed for the primary LASSO model. In each bootstrap sample, the revised primary modeling pipeline, including preprocessing and LASSO logistic regression without class imbalance correction, was refitted and evaluated in both the bootstrap sample and the original cohort to estimate optimism. The previous SMOTE-based pipeline was retained only as a sensitivity analysis. Apparent and optimism-corrected AUC, Brier score, calibration intercept, and calibration slope were reported.

Model calibration was evaluated using calibration plots, Brier scores, calibration intercepts, and calibration slopes. Calibration plots compared predicted probabilities with observed event proportions across quantile-based risk groups and included a smoothed calibration curve when sample size permitted. Because some exploratory external assessment subsets contained very few events, calibration analyses were performed in the most informative available datasets and were interpreted descriptively. In eICU, the PSM-based SLE assessment subset contained only 6 progressive events and was not considered suitable for meaningful calibration assessment. Therefore, eICU calibration was evaluated descriptively in the full eICU SLE assessment cohort using independent LCMM labels. NWICU calibration was similarly evaluated in the available SLE assessment dataset. These calibration analyses were considered complementary to, rather than directly interchangeable with, the PSM-based discrimination analyses.

External trajectory labels were generated according to data structure and longitudinal measurement density. In eICU, sufficient longitudinal creatinine data were available, and independent LCMMs with one to five classes were fitted; the three-class model was used for exploratory external assessment, and classes were named according to the same slope-based mapping procedure as in MIMIC. In NWICU, independent LCMM was not used in the current analysis. Instead, each patient’s relative creatinine change between the first and last available time windows within 7 days was calculated and classified using the MIMIC-derived stable trajectory range. In the local cohort, only 0-, 24-, and 48-hour creatinine values were available, making LCMM unsuitable; therefore, MIMIC-aligned rule-based labeling was used.

To assess the influence of class imbalance handling, we compared three strategies in the MIMIC SLE cohort: no imbalance correction, SMOTE within the training pipeline, and cost-sensitive LASSO logistic regression using class weights. For each strategy, AUC, Brier score, calibration intercept, and calibration slope were reported.

To evaluate the influence of race/ethnicity on model performance and transportability, we performed a sensitivity analysis comparing the original six-variable model with a race-free five-variable model. The race-free model excluded race/ethnicity but otherwise used the same preprocessing, SMOTE, LASSO logistic regression, and performance-assessment procedures. Discrimination and calibration metrics were compared in the MIMIC SLE cohort.

## 3 Results

### 3.1 Characteristics of the MIMIC SLE-AKI cohort

The final MIMIC-IV derivation cohort included 279 patients with SLE-AKI. The mean age was 53.2 ± 16.2 years, and 224 patients (80.3%) were female. Comorbidities were common: hypertension was present in 186 patients (66.7%), chronic kidney disease in 106 patients (38.0%), and heart failure in 103 patients (36.9%). Infection was documented in 169 patients (60.6%). A total of 171 patients (61.3%) received vasopressors, 105 patients (37.6%) received HCQ, and 211 patients (75.6%) received vancomycin. When stratified by AKI trajectory, patients in the progressive group more frequently had hypertension, infection, vasopressor use, and vancomycin exposure. This pattern suggests that the progressive subgroup may have had greater baseline renal vulnerability, hemodynamic instability, and nephrotoxic exposure burden. Complete baseline characteristics are shown in **Table 1**.

**Table 1:** Baseline characteristics of SLE-AKI patients by trajectory class.

Characteristic	Total (n=279)	Progressive (n=26)	Recovery (n=54)	Stable (n=199)	*P*-value
Age, years, mean (SD)	53.2 (16.2)	56.5 (14.8)	52.8 (17.3)	52.9 (16.1)	0.642
Female, n (%)	224 (80.3)	19 (73.1)	42 (77.8)	163 (81.9)	0.489
Race, n (%)					0.312
White	131 (47.0)	9 (34.6)	27 (50.0)	95 (47.7)	
Black	75 (26.9)	9 (34.6)	16 (29.6)	50 (25.1)	
Hispanic	18 (6.5)	3 (11.5)	3 (5.6)	12 (6.0)	
Asian	12 (4.3)	1 (3.8)	2 (3.7)	9 (4.5)	
Other	43 (15.4)	4 (15.4)	6 (11.1)	33 (16.6)	
Hypertension, n (%)	186 (66.7)	22 (84.6)	38 (70.4)	126 (63.3)	0.009
CKD, n (%)	106 (38.0)	11 (42.3)	22 (40.7)	73 (36.7)	0.752
Heart Failure, n (%)	103 (36.9)	12 (46.2)	19 (35.2)	72 (36.2)	0.548
Infection, n (%)	169 (60.6)	20 (76.9)	35 (64.8)	114 (57.3)	0.003
Vasopressors, n (%)	171 (61.3)	24 (92.3)	36 (66.7)	111 (55.8)	<0.001
HCQ Use, n (%)	105 (37.6)	6 (23.1)	22 (40.7)	77 (38.7)	0.264
Vancomycin, n (%)	211 (75.6)	26 (100)	44 (81.5)	141 (70.9)	<0.001

### 3.2 AKI trajectory phenotypes

In the MIMIC SLE-AKI cohort, LCMM identified three clinically interpretable AKI trajectories. Although the four-class and five-class models further reduced AIC and BIC, their minimum class proportions were small, indicating potential instability of small classes and over partitioning. After considering model fit, classification quality, class proportions, and clinical interpretability, the three-class model was selected as the primary trajectory model. This model had an entropy of 0.810 and a mean posterior classification probability of 0.889, indicating good classification quality. Detailed model selection metrics are provided in **Supplementary Table 1**.

The three trajectories were defined as progressive, recovery, and stable. The progressive trajectory included 26 patients (9.3%) and was characterized by a sustained increase in serum creatinine during the first 7 days after AKI onset. The recovery trajectory included 54 patients (19.4%) and was characterized by a high initial creatinine level followed by a marked decline. The stable trajectory included 199 patients (71.3%) and was characterized by relatively stable creatinine values. Class naming was based on the mean direction of creatinine change and posterior classification probabilities; detailed mapping results are shown in **Supplementary Table 2**.

The average creatinine patterns of the three AKI trajectories across different cohorts are shown in **Supplementary Figure 1**. In the MIMIC derivation cohort, the progressive trajectory showed a sustained increase in serum creatinine over 7 days after AKI onset, the recovery trajectory showed a high initial creatinine level followed by rapid improvement, and the stable trajectory remained relatively low and stable. Similar three-class patterns were also observed in the eICU and NWICU assessment cohorts. In eICU, independent LCMM also supported a three-class structure with acceptable classification quality, and the corresponding model-selection metrics are provided in **Supplementary Table 3**. In both external cohorts, the progressive class showed an upward trend, the recovery class showed a downward trend, and the stable class showed only minor fluctuations. These findings suggest that the three-trajectory structure has a degree of reproducibility across databases.

### 3.3 Feature selection and model performance

**Figure 2:**
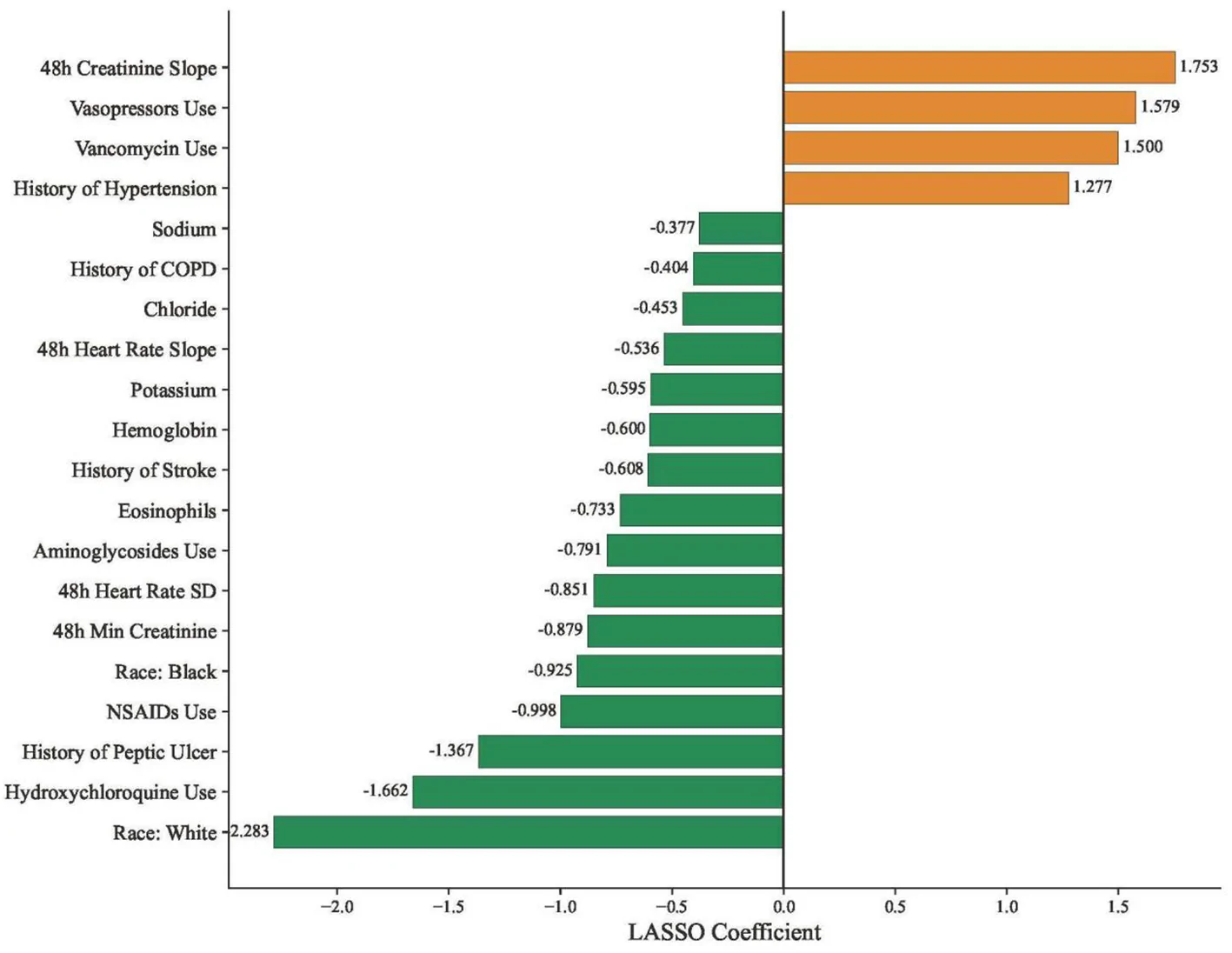
Core feature selection in patients with SLE-AKI. Feature selection was performed using LASSO logistic regression. Positive coefficients indicate increased predicted probability of a progressive AKI trajectory, whereas negative coefficients indicate reduced predicted probability. The 48-hour creatinine slope, vasopressor use, vancomycin use, and history of hypertension were the main risk-direction variables. In the original feature-selection analysis, HCQ use and selected race/ethnicity categories showed protective-direction coefficients. However, race/ethnicity was excluded from the revised primary model and retained only in secondary sensitivity analyses. The revised primary model selected five core variables based on LASSO results, clinical interpretability, calibration, and cross-database transportability. Race/ethnicity was excluded from the primary model.

**Figure 3:**
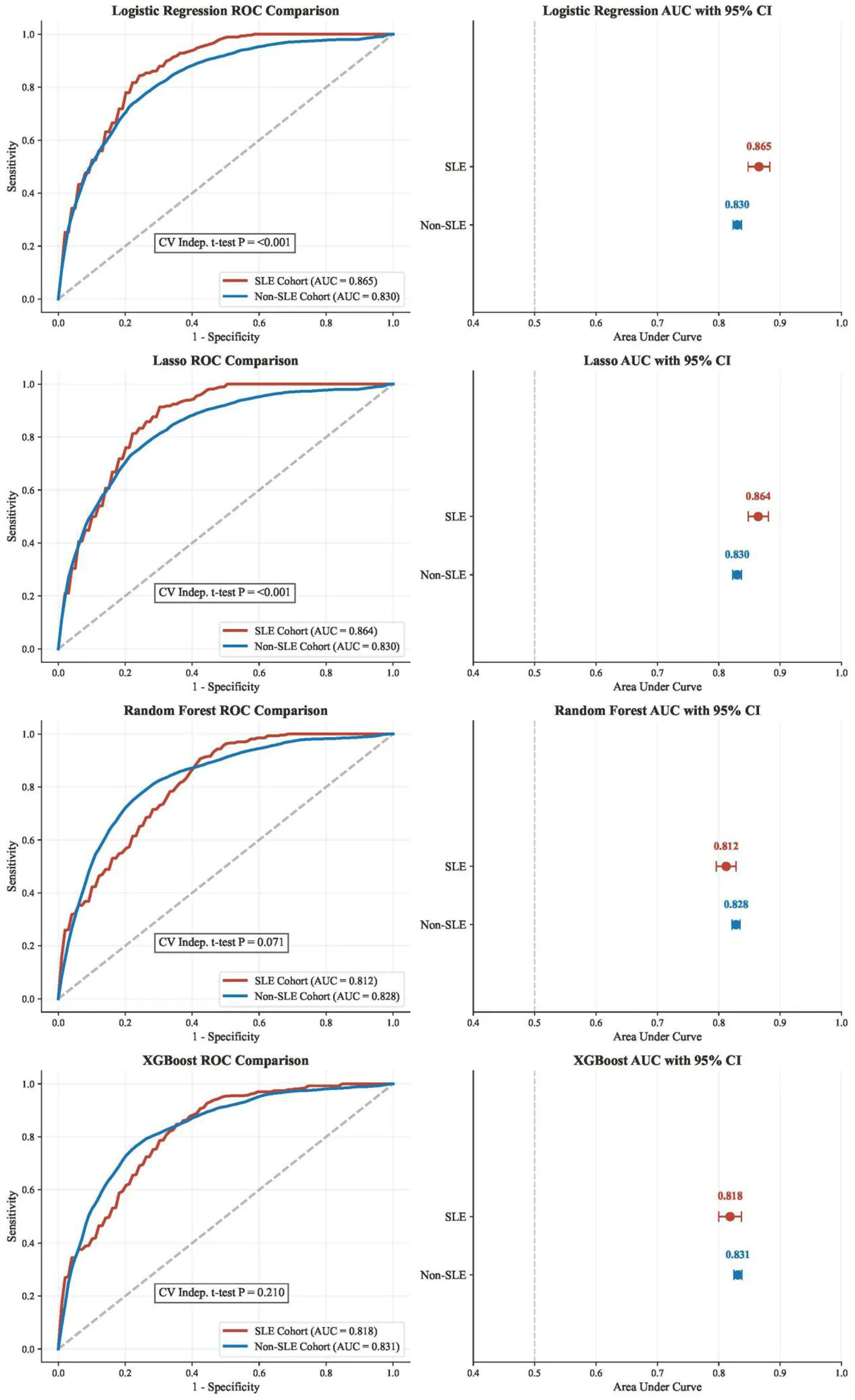
ROC comparison of different models in the MIMIC cohort. The image compares the risk-ranking performance of standard logistic regression, LASSO, random forest, and XGBoost in the SLE and non-SLE AKI cohorts. LASSO and standard logistic regression achieved the best performance in the SLE cohort and outperformed random forest and XGBoost.

LASSO feature selection showed that the 48-hour creatinine slope, vasopressor use, vancomycin use, and history of hypertension were associated with a higher risk of progressive AKI, whereas HCQ use was associated with a lower risk of progression (**Figure 2**). Based on LASSO results, clinical interpretability, calibration, and cross-database transportability, the revised primary risk-stratification model retained five core variables: 48-hour creatinine slope, vasopressor use, history of hypertension, HCQ use, and vancomycin use. Race/ethnicity was excluded from the primary model and evaluated only in a secondary sensitivity analysis. To compare feature selection patterns between SLE and non-SLE AKI populations, a LASSO analysis was also performed in the non-SLE AKI cohort. In the non-SLE cohort, the 48-hour creatinine slope remained the strongest positive predictor. Diuretic use, vasopressor use, vancomycin use, coronary artery disease, infection, and chronic kidney disease also showed positive predictive directions. In contrast, HCQ use did not emerge as a major feature in the non-SLE cohort, suggesting that its negative predictive contribution may be more relevant in the SLE-AKI context (**Supplementary Figure 2**). Among the four modeling approaches, LASSO and standard logistic regression showed better risk-ranking performance than random forest and XGBoost in the MIMIC SLE cohort (**Figure 3**).

The revised primary race-free, no-imbalance-correction LASSO model included five predictors: 48-hour creatinine slope, vasopressor use, hypertension history, HCQ use, and vancomycin use. The model achieved an apparent AUC of 0.761, a Brier score of 0.086, a calibration intercept of 0.086, and a calibration slope of 1.353. After bootstrap optimism correction (1000 resamples), the corrected AUC was 0.706, the corrected Brier score was 0.092, the corrected calibration intercept was -0.054, and the corrected calibration slope was 0.947 (**Supplementary Table 6**). For comparison, the previously reported SMOTE-based six-variable model (including race/ethnicity) yielded a repeated cross-validation AUC of 0.870 but had less favorable calibration (Brier score 0.179). A non-overlap sensitivity analysis assessed whether the primary model risk score discriminated a post-48-hour hard renal endpoint (creatinine doubling or death) that did not incorporate creatinine values from the predictor window. Discrimination was substantially attenuated (AUC 0.593, Brier 0.102), indicating that a considerable portion of the model’s apparent discrimination reflected structural overlap between the 48-hour creatinine slope and the 7-day trajectory-defined outcome. The previously reported cross-validated AUC of 0.870 for the six-variable race-including SMOTE-based model is included in **Supplementary Table 8**, whereas race-free imbalance-handling comparisons are summarized in **Supplementary Table 9**.

The derivation cohort contained 26 progressive events, yielding an EPV of 5.2 for the revised primary five-variable model. For the previous six-variable race-including sensitivity model, the EPV was 4.3. External calibration assessment was descriptive because of small event counts. A non-overlap sensitivity analysis showed diminished discrimination (AUC 0.593) when assessed against an independent post-48-hour endpoint.

Calibration analysis of the primary LASSO model across cohorts, using the calibration analysis datasets described in the Methods, is summarized in **Supplementary Table 7** and **Supplementary Figure 9**. In the MIMIC SLE cohort, the revised primary model showed a Brier score of 0.086, a calibration intercept of 0.086, and a calibration slope of 1.353. In eICU, the uncalibrated MIMIC-trained model showed limited discrimination in the full SLE calibration dataset using independent LCMM labels, with an AUC of 0.551. Predicted probabilities were severely shifted upward, with a median predicted probability of 0.999 and a Brier score of 0.737. Therefore, the eICU Brier score was interpreted as evidence of uncalibrated probability shift rather than meaningful external calibration. In NWICU and the local cohort, calibration metrics were considered exploratory because of very small event counts.

### 3.4 Associations between core variables and AKI progression

**Figure 4:**
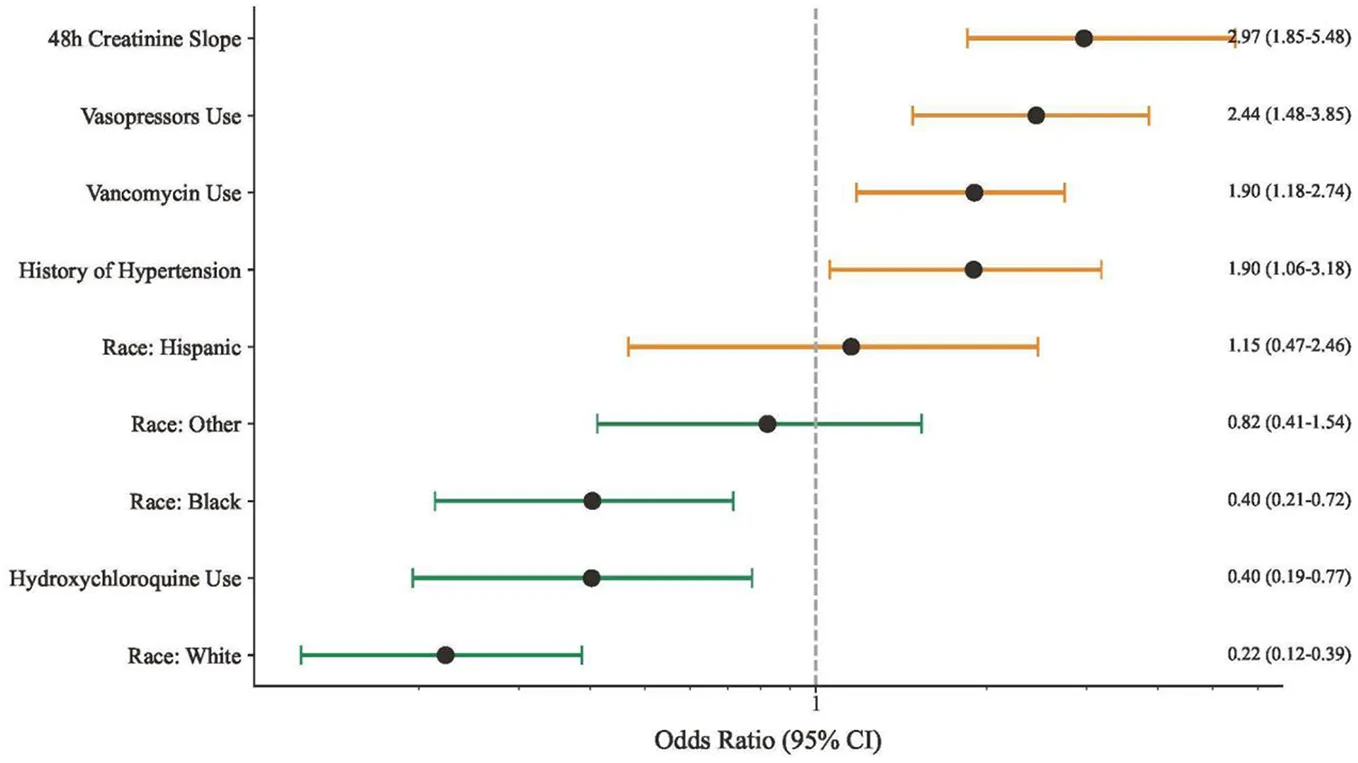
Independent factors associated with AKI progression in patients with SLE-AKI. The forest plot shows the ORs and 95% CIs of core variables in the multivariable logistic regression model. The 48-hour creatinine slope, vasopressor use, vancomycin use, and history of hypertension were associated with an increased risk of progressive AKI, whereas HCQ use was associated with lower odds of progression in this observational multivariable model.

In the SLE-AKI cohort, a multivariable logistic regression model based on the previous six-variable race-including model (retained as a sensitivity analysis) was fitted to assess their independent associations with the progressive AKI trajectory (**Figure 4**). The 48-hour creatinine slope had the strongest association with AKI progression, with an OR of 2.97 (95% CI, 1.85–5.48). Vasopressor use was associated with an increased risk of progression, with an OR of 2.44 (95% CI, 1.48–3.85). Vancomycin use was also associated with a higher risk of AKI progression, with an OR of 1.90 (95% CI, 1.18–2.74). A history of hypertension was similarly associated with increased progression risk, with an OR of 1.90 (95% CI, 1.06–3.18). HCQ use showed an observational association with lower odds of AKI progression, with an OR of 0.40 (95% CI, 0.19–0.77). Certain race/ethnicity categories showed a lower-risk direction in the model; however, these findings may reflect social determinants, access to care, coding practices, cohort composition, and residual confounding rather than biological causality.

**Figure 5:**
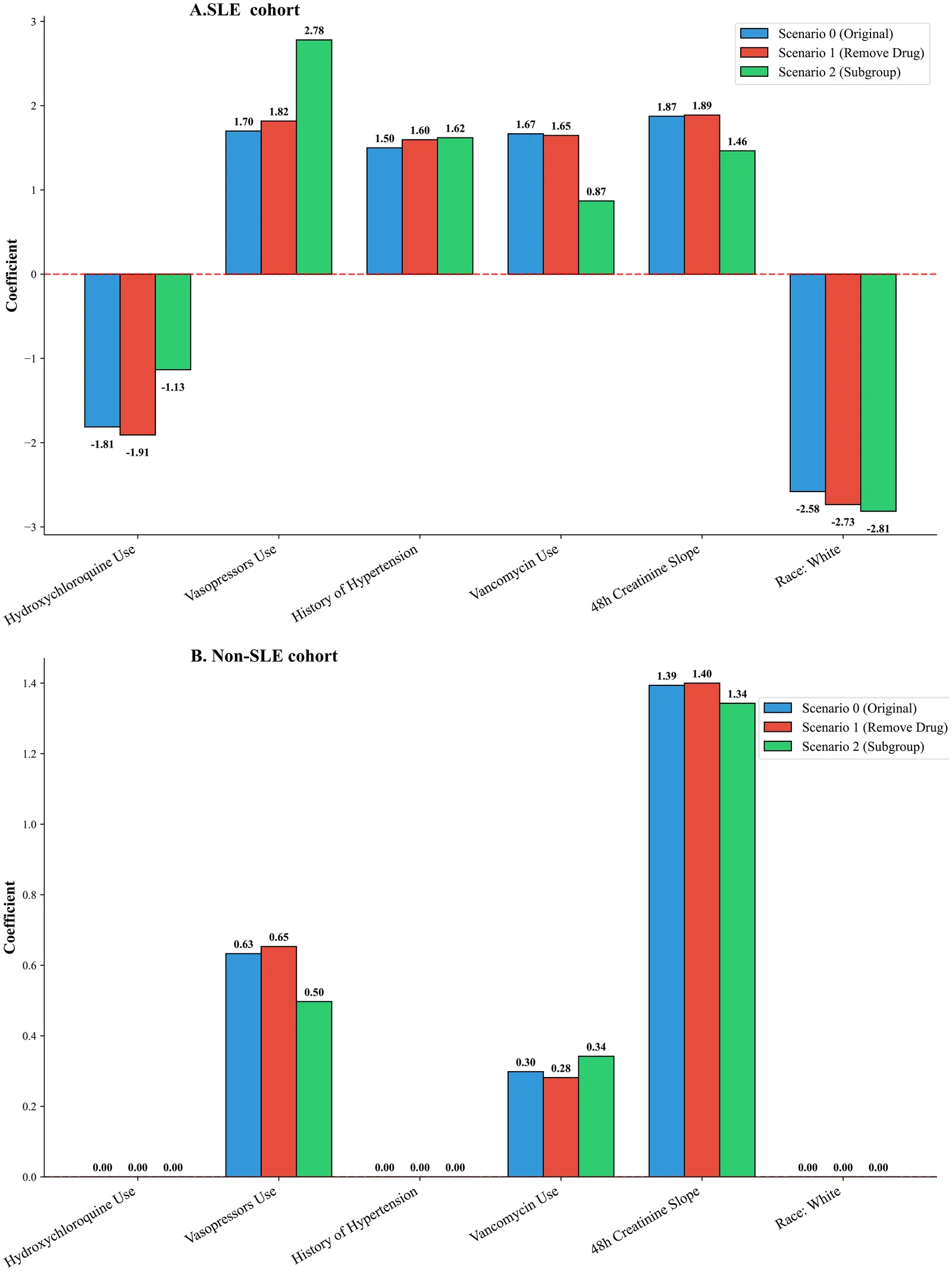
Sensitivity analysis of core feature coefficient stability. **(A)** SLE cohort. **(B)** Non-SLE cohort. The image shows coefficient changes of core features across different modeling scenarios in each cohort. HCQ use consistently showed a negative coefficient in the SLE cohort but had a coefficient close to zero in the non-SLE cohort. The 48-hour creatinine slope remained a stable positive predictor in both cohorts.

Sensitivity analyses of core feature stability showed that HCQ use consistently retained a negative coefficient across different modeling scenarios in the SLE cohort (**Figure 5**). The HCQ coefficient was approximately −1.81 in the primary analysis, −1.91 after removing selected medication variables, and −1.13 in the prespecified subgroup analysis. The 48-hour creatinine slope, vasopressor use, hypertension, and vancomycin use generally maintained positive coefficients across modeling scenarios. White race/ethnicity generally retained a negative coefficient. In the non-SLE cohort, the 48-hour creatinine slope remained the most stable positive predictor, whereas the coefficient for HCQ use was zero or close to zero. These findings support the observation that the protective direction of HCQ in the SLE cohort was not entirely driven by the general AKI risk structure, although indication bias, treatment selection bias, and residual confounding cannot be excluded.

Although HCQ use did not differ significantly across trajectory classes in univariate comparison, it was retained in the revised primary risk-stratification model because it showed a non-zero LASSO coefficient, was clinically relevant to SLE management, and was available across assessment datasets. This discrepancy underscores that univariate comparisons may not capture conditional associations after adjustment for hemodynamic instability, nephrotoxic exposure, hypertension, and early creatinine dynamics. Race/ethnicity-adjusted analyses were retained only as secondary sensitivity analyses.

**Figure 6:**
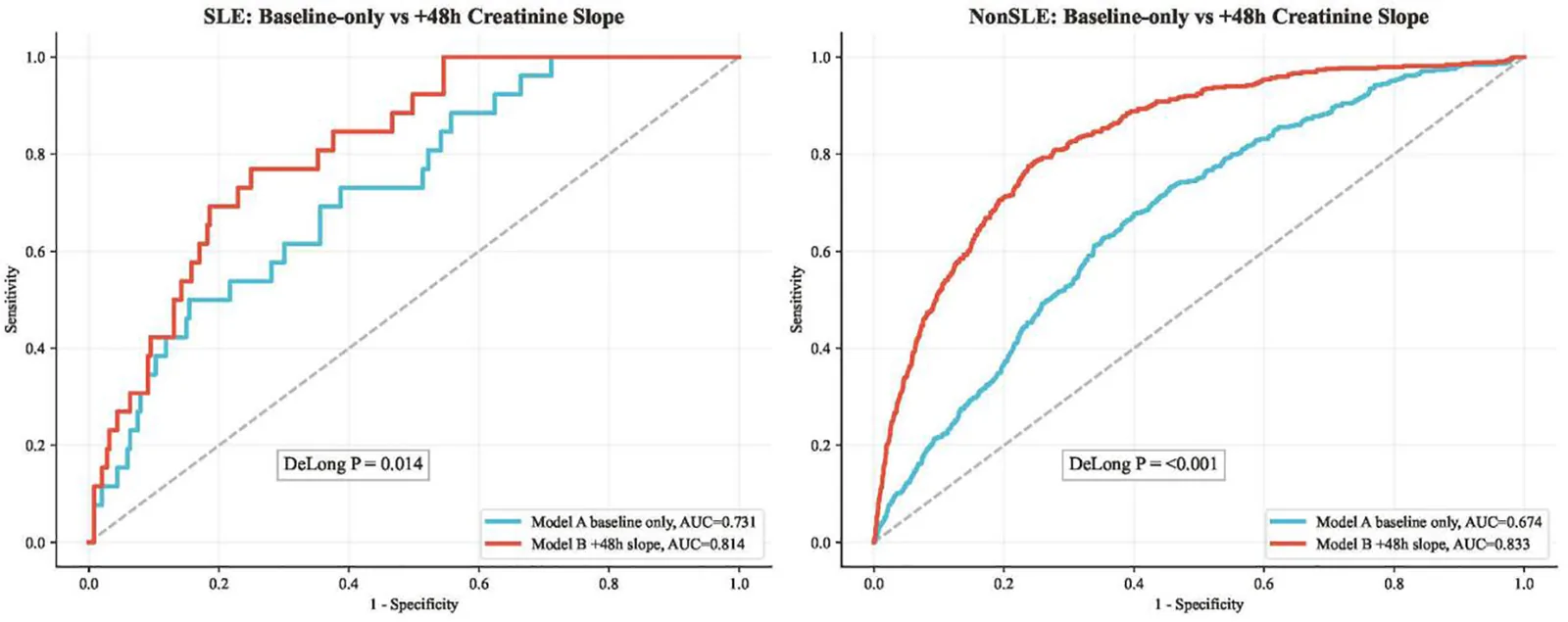
Incremental risk-ranking contribution of the 0–48-hour creatinine slope. The image compares ROC curves between the baseline-variable model and the model incorporating the 48-hour creatinine slope. In the SLE cohort, adding the 48-hour creatinine slope increased the AUC from 0.731 to 0.814. In the non-SLE cohort, the AUC increased from 0.674 to 0.833, indicating that early dynamic changes in serum creatinine substantially improved risk-ranking performance.

The incremental risk-ranking contribution of the 48-hour creatinine slope was further evaluated (**Figure 6**). In the SLE cohort, the AUC of the model using baseline variables alone was 0.731 and increased to 0.814 after adding the 48-hour creatinine slope (P = 0.014). In the non-SLE cohort, the AUC increased from 0.674 to 0.833 after adding the creatinine slope (P<0.001). These findings indicate that early dynamic changes in serum creatinine after AKI onset are important for risk-ranking subsequent trajectory patterns. They also suggest that the proposed model is best positioned as a dynamic risk-updating tool within the first 48 hours after AKI onset rather than as an immediate risk-stratification model at hospital admission.

### 3.5 HCQ use and AKI progression

To further evaluate the observational association between HCQ use and AKI progression, propensity score matching was performed within the SLE-AKI cohort (**Figure 1**). After matching, the AKI progression rate was 11.5% in the non-HCQ group and 1.6% in the HCQ group, with a borderline between-group difference by chi-square testing (P = 0.067). In a bootstrap exploratory logistic model including HCQ exposure only, the estimated OR was 0.333 (95% CI, 0.175–0.777). Given the small matched sample and low number of events, these findings were interpreted as hypothesis-generating rather than confirmatory. In the combined SLE and non-SLE cohort, the SLE × HCQ interaction term did not reach statistical significance (**Supplementary Figure 3**).

### 3.6 Exploratory external assessment

Given the very small number of external progressive events and the use of cohort-specific outcome-labeling procedures, all external cohort analyses were interpreted as exploratory external assessments rather than definitive validation. Accordingly, AUC estimates across cohorts should not be directly compared, pooled, or ranked.

In the eICU exploratory external assessment cohort, after propensity score matching, the SLE cohort included 94 patients, of whom 6 had progressive events; the non-SLE cohort included 282 patients, of whom 22 had progressive events. In the eICU SLE cohort, the LASSO model achieved an AUC of 0.797 (95% CI, 0.608–0.946), compared with 0.680 (95% CI, 0.530–0.817) in the non-SLE cohort (P = 0.306; **Supplementary Table 4**; **Supplementary Figure 4**). Standard logistic regression yielded an AUC of 0.725 (95% CI, 0.548–0.894) in the SLE cohort and 0.679 (95% CI, 0.522–0.814) in the non-SLE cohort (P = 0.691). Random forest yielded an AUC of 0.745 (95% CI, 0.488–0.943) in the SLE cohort and 0.706 (95% CI, 0.568–0.842) in the non-SLE cohort (P = 0.782). XGBoost yielded an AUC of 0.734 (95% CI, 0.531–0.902) in the SLE cohort and 0.718 (95% CI, 0.576–0.841) in the non-SLE cohort (P = 0.894). These eICU exploratory assessment results suggest that the LASSO model retained moderate risk-ranking ability in an external SLE cohort, although performance was lower than that observed in the MIMIC internal assessment.

In the NWICU exploratory external assessment cohort, after propensity score matching, the SLE cohort included 49 patients, of whom 3 had progressive events; the non-SLE cohort included 140 patients, of whom 6 had progressive events. In the SLE cohort, the LASSO model achieved an AUC of 0.964 (95% CI, 0.891–1.000), compared with 0.831 (95% CI, 0.634–0.973) in the non-SLE cohort (P = 0.135; **Supplementary Table 5**; **Supplementary Figure 5**). Standard logistic regression yielded an AUC of 0.964 (95% CI, 0.881–1.000) in the SLE cohort and 0.821 (95% CI, 0.623–0.973) in the non-SLE cohort (P = 0.126). Random forest yielded an AUC of 0.848 (95% CI, 0.563–1.000) in the SLE cohort and 0.762 (95% CI, 0.481–0.971) in the non-SLE cohort (P = 0.632). XGBoost yielded an AUC of 0.928 (95% CI, 0.813–1.000) in the SLE cohort and 0.790 (95% CI, 0.533–0.978) in the non-SLE cohort (P = 0.274). These NWICU results showed numerically high AUCs, particularly for the linear models. However, because the NWICU SLE cohort included only three progressive events, these estimates are highly unstable and should be interpreted with caution.

In the local 18-patient exploratory external assessment cohort, the primary LASSO model achieved an exploratory continuous-probability AUC of 0.824 and a Brier score of 0.124. The only progressive patient had a predicted probability of 0.529. However, two non-progressive patients had predicted probabilities of 0.568, which were higher than that of the progressive patient, and several additional non-progressive patients had probabilities close to the progressive case. Therefore, the local exploratory assessment suggested partial risk separation but not perfect ranking or perfect classification. Given the extremely small sample size and the presence of only one progressive event, these findings were interpreted as exploratory (**Supplementary Figures 6**–**9**).

In the race-free model comparison, exclusion of race/ethnicity resulted in an AUC of 0.845 in the MIMIC SLE cohort, compared with 0.870 for the original six-variable model. The corresponding Brier scores were 0.192 and 0.179, and calibration slopes were 1.323 and 1.355, respectively (**Supplementary Table 8**; **Supplementary Figure 10**). These findings suggest that race/ethnicity contributed limited incremental predictive value in the MIMIC SLE cohort.

In the imbalance-handling sensitivity analysis based on the race-free predictor set, the SMOTE-based LASSO model achieved a higher AUC than the no-correction model (0.845 vs. 0.761), whereas the no-correction model had more favorable calibration, with a lower Brier score (0.086 vs. 0.192) and a calibration intercept closer to zero (0.086 vs. -1.142; **Supplementary Table 9**). The previous six-variable race-including SMOTE model achieved an AUC of 0.870 and is reported separately as a secondary sensitivity analysis in **Supplementary Table 8**.

### 3.7 Online risk-stratification calculator

As an exploratory supplementary resource, we implemented a web-based calculator using the revised primary risk-stratification framework. The calculator defaults to the revised five-variable race-free model and returns an estimated probability of progressive trajectory membership. It is available at https://sle-aki-trajectories.vercel.app/. This tool is intended for research demonstration and prospective testing only and should not be used to guide clinical decision-making before prospective validation and local recalibration.

## 4 Discussion

Using multidata base electronic health record data, this study identified clinically interpretable AKI trajectory phenotypes and developed an exploratory, interpretable early risk-stratification framework for progressive trajectory membership in patients with SLE-AKI. We first applied LCMM to identify three AKI trajectories—progressive, recovery, and stable—highlighting the substantial heterogeneity of renal function evolution after AKI in patients with SLE-AKI (34). We then constructed a parsimonious five-variable, race-free risk-stratification model based on LASSO feature selection and clinical interpretability. The model showed moderate apparent discrimination in MIMIC, with attenuation after bootstrap optimism correction and in the non-overlap sensitivity analysis. In addition, HCQ use showed an exploratory association with lower odds of AKI progression, although its potential disease specificity and causal relevance require further validation.

The identification of three AKI trajectories has important clinical implications. Although the progressive trajectory accounted for only 9.3% of the MIMIC SLE-AKI cohort, it represents the high-risk subgroup for whom early recognition is most clinically relevant. Patients in this group more frequently had hypertension, infection, vasopressor use, and vancomycin exposure, suggesting that AKI progression may be driven by a combination of baseline renal vulnerability, hemodynamic instability, infection-related inflammation, and nephrotoxic drug exposure. Compared with conventional AKI staging, trajectory-based phenotyping more directly captures the direction and speed of renal function change over time and may provide a more informative basis for risk stratification and intervention timing (5–7).

In this study, LASSO and standard logistic regression outperformed random forest and XGBoost. Although complex machine learning algorithms can be advantageous in many high-dimensional medical data settings (19), their utility may be limited when the number of outcome events is small and the relationships between core predictors and outcome are clinically coherent. In such settings, complex models may be more vulnerable to noise and sample-size constraints. By contrast, parsimonious linear models offer fewer variables, clear coefficient directions, strong interpretability, low implementation burden, and easier external assessment. In high-risk medical decision-making contexts, interpretability is itself an important attribute (20, 21). Accordingly, a regularized logistic regression framework was considered appropriate as the primary translational risk-stratification model in this study.

This study also contributes to the broader discussion regarding interpretable versus black-box AI in high-stakes clinical risk stratification. In a setting with only 26 progressive events, complex ensemble methods did not clearly outperform parsimonious linear models. This finding is consistent with the view that clinical AI tools should not be judged by discrimination alone, but also by interpretability, calibration, reproducibility, transportability, and clinical plausibility. For complex autoimmune diseases such as SLE, where unmeasured disease activity and treatment context strongly influence outcomes, transparent models may be more appropriate for early translational work than black-box models.

The 48-hour creatinine slope was the most important and stable predictor in our analysis. It was consistently selected by LASSO, remained strongly associated with AKI progression in multivariable logistic regression, and substantially improved the risk-ranking performance of baseline models. AKI is inherently a dynamic process, and a single creatinine measurement often cannot distinguish patients who are recovering from those who are continuing to deteriorate. The 0–48-hour creatinine slope captures early injury velocity or recovery tendency and is therefore more closely aligned with the goal of trajectory risk stratification than static variables alone. Importantly, a model incorporating the 48-hour creatinine slope is not an admission-time prediction model. Rather, it should be considered a dynamic risk-updating tool within the first 48 hours after AKI onset. Such a tool could help clinicians identify patients who may continue to worsen and may benefit from intensified monitoring, optimization of hemodynamic management, cautious use of nephrotoxic agents, and early nephrology consultation (22–24).

The association between HCQ use and a lower risk of AKI progression is another notable finding. Both multivariable logistic regression and propensity score matching suggested an exploratory association between HCQ use and lower odds of the progressive AKI trajectory. Sensitivity analyses further showed that HCQ had a consistently protective direction in the SLE cohort but contributed little or nothing in the non-SLE cohort. This observation is broadly consistent with previous evidence linking HCQ use to improved systemic and renal outcomes in SLE, although the present study was not designed to test a causal protective effect (25, 26). Potential mechanisms include reduced SLE disease activity, attenuation of immune complex–mediated injury, modulation of inflammatory responses, and long-term renal protection (27–29). Nevertheless, this study cannot establish a causal protective effect of HCQ on AKI progression. Patients receiving HCQ may have had more standardized disease management, better long-term follow-up, greater treatment adherence, or different baseline disease characteristics.

Although HCQ use showed a consistent negative association with progressive AKI in multivariable modeling and propensity score analysis, these results should not be interpreted as evidence of a causal protective effect. The PSM analysis included only 61 matched pairs, the between-group comparison was borderline significant, and residual confounding by disease activity, treatment adherence, access to care, and unmeasured SLE-specific variables remains likely. Therefore, the present study does not establish a causal protective effect of HCQ on AKI progression.

The exploratory external assessment results provide preliminary information about model transportability but also highlight substantial cross-database heterogeneity. In eICU, the LASSO model achieved an AUC of 0.797 in the SLE cohort, suggesting moderate risk-ranking ability in this exploratory assessment. However, this estimate was based on only 6 progressive events and should not be interpreted as definitive evidence of external generalizability. This decline may reflect differences in patient characteristics, testing frequency, data recording practices, trajectory labeling methods, and medication variable completeness. In particular, HCQ exposure data may be less reliable in some external databases, and HCQ was one of the core variables in the final model. This limitation may affect model transferability in patients with SLE. In NWICU, the model produced numerically high AUCs, but the SLE cohort included only three progressive events and the confidence intervals were wide. Similarly, the local assessment cohort of 18 patients showed partial but imperfect risk separation. The only progressive patient received an elevated predicted probability, but several non-progressive patients had similar or higher predicted probabilities. Therefore, with only one positive event, the local assessment results should be interpreted as exploratory feasibility evidence rather than as definitive evidence of discrimination or classification performance. Overall, the exploratory external assessments provide preliminary information about model transportability, but they do not establish generalizability. Larger multicenter cohorts with more events and more complete medication data are required for definitive evaluation of generalizability (30, 31).

The calibration analyses further highlight the distinction between risk ranking and absolute risk prediction. Although the model showed moderate-to-high risk-ranking performance in some exploratory analyses, predicted probabilities were not consistently transportable across databases. This was most evident in eICU, where the MIMIC-trained model assigned very high predicted probabilities to most patients, resulting in a markedly elevated Brier score. This finding suggests substantial probability shift due to differences in case mix, outcome-label construction, measurement density, medication recording, and database structure. Therefore, the current model should be interpreted primarily as a risk-ranking tool, and local recalibration or model updating would be required before using predicted probabilities for individual-level clinical decision-making.

The class-imbalance sensitivity analysis also showed that discrimination and calibration may move in opposite directions. SMOTE improved AUC but produced a less favorable Brier score than the unweighted model, suggesting that oversampling may distort predicted probabilities. Therefore, the SMOTE-based model may be more reliable for identifying higher-risk patients than for providing precise absolute risk estimates. Before clinical deployment, recalibration in the target population would likely be necessary.

Another important limitation is that trajectory labels were not generated identically across all external cohorts. eICU used independent LCMM labels, whereas NWICU and the local cohort used MIMIC-aligned rule-based labels. These approaches were chosen according to longitudinal creatinine density and sample size, but they may introduce outcome-label heterogeneity and influence external AUC and calibration estimates. Cross-cohort differences in model performance should therefore be interpreted as evidence of model behavior under realistic data heterogeneity rather than as directly interchangeable validation estimates.

This study has potential clinical translational value. The revised primary model requires only five routinely available clinical variables. The 48-hour creatinine slope can be calculated from routine serum creatinine measurements, while vasopressor use, vancomycin use, HCQ use, and hypertension history can be obtained from electronic health records. The race-including model is retained only as a secondary research comparison. The online calculator further enhances the model’s testability. In the future, this tool may support dynamic risk assessment within 48 hours after AKI onset and help identify patients with SLE-AKI who require closer monitoring or early intervention. Before routine clinical deployment, however, prospective validation, workflow integration testing, model calibration assessment, and clinical utility evaluation will be necessary.

The exploratory web-based calculator should also be interpreted cautiously. It has not undergone formal interface validation, calibration updating, workflow integration testing, or clinical impact evaluation. Therefore, it should not be used to guide treatment decisions outside a research setting.

Several limitations should be acknowledged. First, the retrospective design precludes causal inference, particularly regarding the association between HCQ use and AKI progression, which may be affected by indication bias and residual confounding. Second, SLE diagnosis, AKI identification, and medication exposure were all derived from electronic health record data and may therefore be subject to misclassification. Third, publicly available ICU databases lack complete information on SLE disease activity, complement levels, anti-dsDNA antibodies, proteinuria, urine sediment, renal biopsy class, and immunosuppressive regimens. As a result, these important disease-specific variables could not be incorporated into the model. Fourth, LCMM trajectory labels were data-driven; although the trajectory shapes were clinically interpretable, classification error remains possible. Fifth, the MIMIC SLE-AKI cohort included only 26 progressive cases, limiting model complexity and precluding robust three-class prediction. Sixth, the number of events in the exploratory external assessment cohorts was limited, particularly in NWICU and the local assessment cohort, making AUC and confusion matrix estimates unstable. Seventh, race/ethnicity contributed to the previous race-including sensitivity model, but it was excluded from the revised primary model because it may reflect social determinants, healthcare access, coding differences, structural inequities, and database composition rather than biological causal effects.

The most important modeling limitation is the small number of progressive events. The MIMIC SLE-AKI cohort included only 26 progressive cases, corresponding to an EPV of 5.2 for the revised primary five-variable model. Although this is higher than the EPV of the previous six-variable model, it remains low and increases the risk of overfitting, coefficient instability, and imprecise association estimates. Repeated cross-validation and bootstrap optimism correction help quantify this risk but cannot eliminate it. Therefore, the present model should be considered an early parsimonious risk-stratification framework requiring prospective validation, recalibration, and possible model updating in larger cohorts.

The treatment of race/ethnicity deserves particular caution. Race/ethnicity should not be interpreted as a biological determinant of AKI progression. Rather, it may encode social determinants, healthcare access, structural inequities, coding practices, and cohort composition. For this reason, race/ethnicity was excluded from the revised primary model. The previous race-including six-variable model was retained only as a secondary sensitivity analysis to document the effect of excluding race/ethnicity on discrimination and calibration.

A further limitation is the absence of key SLE-specific disease activity variables in ICU databases, including complement levels, anti-dsDNA antibody titers, proteinuria, urine sediment, renal biopsy class, SLE disease activity scores, glucocorticoid dose, and immunosuppressive regimens. These variables are central to SLE-AKI pathophysiology and may confound both AKI trajectory risk and HCQ prescribing. Their absence may reduce generalizability to rheumatology-specific settings where disease activity data are available. Future model updating should incorporate these variables or EHR-derived proxies such as steroid dose, immunosuppressant exposure, nephrology or rheumatology consultation, and proteinuria measurements.

In summary, this study shows that progressive AKI trajectories in patients with SLE-AKI can be risk-stratified using early post-AKI creatinine dynamics and a small number of routinely available clinical variables. HCQ use showed a hypothesis-generating observational association with lower odds of AKI progression, but this finding should not be interpreted as causal. Exploratory external assessment in the eICU, NWICU, and local cohorts provided preliminary evidence of model transportability, although performance was influenced by database heterogeneity, trajectory labeling methods, medication data completeness, and event numbers. The online risk calculator provides a basis for future prospective validation and clinical decision-support system development, but larger prospective multicenter studies with sufficient events are needed before formal clinical implementation.

In conclusion, this study identifies clinically interpretable AKI trajectories in patients with SLE-AKI using latent class mixed modeling and provides an exploratory, race-free risk-stratification framework based on early post-AKI creatinine dynamics. However, the apparent discrimination was substantially attenuated after bootstrap optimism correction and after removing the structural predictor-outcome overlap. External cohort assessments were event-limited and used heterogeneous outcome-labeling schemes; therefore, they should be interpreted as exploratory transportability assessments rather than definitive evidence of generalizability. The model should be viewed as a preliminary risk-stratification tool requiring prospective validation, local recalibration, and the use of independent non-overlapping endpoints before any clinical application. The observed association between HCQ exposure and lower odds of a progressive trajectory is exploratory and hypothesis-generating, not causal.

## Data Availability

The raw data supporting the conclusions of this article will be made available by the authors, without undue reservation.
