# Supplementary material for: Early creatinine dynamics for stratifying acute kidney injury trajectories in patients with systemic lupus erythematosus: an exploratory multicenter retrospective study
Source: Front Immunol. 2026 Aug 17;17:1910588. doi: 10.3389/fimmu.2026.1910588 (PMC13527002; doi:10.3389/fimmu.2026.1910588)
Supplement: Supplementary file 1 [file Supplementaryfile1.docx]

## Supplementary Methods

### Study Workflow and Overall Analytical Framework

The analytical workflow of this study was implemented using one R script and four Python scripts. The R script was primarily used for longitudinal serum creatinine trajectory modeling, LCMM trajectory label generation, and rule-based label construction for the eICU, NWICU, and local assessment cohorts. The main Python analysis script was used for feature selection, risk factor analysis, predictive model training, internal cross-validation, sensitivity analyses, interaction analysis, and model parameter export in the MIMIC cohort. Three additional exploratory external assessment scripts were used to assess model transportability in the eICU, NWICU, and local 18-patient cohorts.

The MIMIC cohort served as the model development and internal assessment cohort. The eICU, NWICU, and local 18-patient cohorts served as exploratory external assessment cohorts. The primary risk-stratification target was membership in the progressive AKI trajectory. The binary outcome variable, target, was defined as follows: progressive trajectory was coded as 1, whereas stable and recovery trajectories were combined and coded as 0.

### Cleaning of Longitudinal Creatinine Data in the MIMIC Cohort

Before analysis, stay_id and time_window were converted into numeric variables. Only creatinine records obtained within 7 days after AKI onset were retained, namely records with time_window ≤7, and records with missing creatinine values were removed. To ensure a minimum amount of longitudinal information for trajectory modeling, only patients with at least three serum creatinine measurements within 7 days after AKI onset were included.

Creatinine values used for LCMM were log-transformed as log_cr = log(creatinine + 0.01). The constant 0.01 was added to avoid failure of log transformation caused by extremely low or anomalous zero values. For trajectory visualization, original creatinine values were used to facilitate clinical interpretation.

### LCMM Trajectory Modeling in the MIMIC Cohort

Post-AKI serum creatinine trajectories were identified using latent class mixed models. LCMM was implemented using the hlme function from the R package lcmm. A one-class model was first fitted with log_cr ~ time_window as the fixed effect, a random intercept as the random effect, and stay_id as the subject identifier. Multiclass models with two to five classes were then fitted based on the one-class model. The fixed effect remained time_window, the class-specific mixed effect was specified as mixture = ~ time_window, the random effect was a random intercept, and the class membership model was specified as an intercept-only model, classmb = ~ 1.

To reduce the influence of local optima, models with two to five classes were fitted using gridsearch with multiple initial values. For each model, 30 random initial value searches were conducted with 20 preliminary iterations, using the one-class model as the initial model. Fitted models with different numbers of classes were saved as RDS files to support review and reproducibility.

Model selection was based on log-likelihood, AIC, BIC, entropy, minimum class proportion, mean posterior classification probability, and convergence status. The final script selected the three-class LCMM as the primary trajectory model and exported each patient’s class assignment and posterior probability. Of note, the LCMM used in the current code included a linear time term only; quadratic terms, spline terms, and random slopes were not used.

### LCMM Trajectory Naming and Binary Outcome Construction

The three classes generated by LCMM were initially represented by numeric class labels. To convert statistical classes into clinically interpretable trajectory phenotypes, the script first calculated the mean original serum creatinine value at each time_window within each LCMM class. The creatinine change from the first to the last available time window was then calculated for each class.

The class with the largest mean increase in creatinine was labeled progressive, the class with the largest mean decrease was labeled recovery, and the remaining class was labeled stable. A three-category label, target_label, was then generated, followed by a binary outcome variable, target. Progressive was coded as 1, while stable and recovery were coded as 0.

### Trajectory Label Generation in the eICU Cohort

In the eICU cohort, LCMMs with one to five classes were also fitted, and the three-class model was ultimately used for sensitivity analysis. Class naming followed the same procedure as in the MIMIC cohort. Specifically, classes were labeled progressive, recovery, or stable according to the direction and magnitude of the mean creatinine change between the first and last time windows.

### Trajectory Label Generation in the NWICU Cohort

Longitudinal creatinine data for the NWICU cohort were obtained from longitudinal_cr_nwicu.csv, and feature data were obtained from SLE_aki_features_nwicu.csv. The script retained creatinine records within 7 days after AKI onset with time_window >0, removed missing creatinine values, and required each patient to have at least three serum creatinine measurements.

A separate LCMM was not fitted in the NWICU cohort. Instead, rule-based trajectory labels were generated using the stable trajectory range derived from MIMIC. For each patient, the relative creatinine change between the first and last available time windows was calculated. Patients whose relative creatinine increase exceeded the upper bound of the MIMIC stable range were labeled progressive. Patients whose relative creatinine change was below the lower bound of the MIMIC stable range were labeled recovery. All others were labeled stable. The resulting trajectory labels were then merged with the NWICU feature table by stay_id to generate the NWICU exploratory external assessment dataset.

### Trajectory Label Generation in the Local 18-Patient Assessment Cohort

The local assessment cohort consisted of two tables: a baseline feature table and a creatinine time-point table. Creatinine time points included SCr_0h, SCr_24h, and SCr_48h. The script first transformed the wide-format creatinine data into long format. The 0-hour, 24-hour, and 48-hour measurements were mapped to time_window values of 1, 2, and 3 for ordering purposes, while the true time variable time_hour was retained as 0, 24, and 48.

Because the local assessment cohort included only 0–48-hour creatinine data rather than complete 7-day longitudinal creatinine sequences, LCMM was not fitted in this cohort. Instead, rule-based labels were assigned using the MIMIC stable trajectory reference range. Specifically, each patient’s relative creatinine change from 0 to 48 hours was calculated. Patients exceeding the MIMIC stable upper bound were labeled progressive, patients below the stable lower bound were labeled recovery, and the remainder were labeled stable. The 48-hour creatinine slope was calculated by linear regression using the 0-, 24-, and 48-hour measurements. If at least two creatinine time points were available, linregress was used to calculate the slope; if fewer than two points were available, the slope was set to 0.

### Variable Preprocessing and Feature Encoding

Race/ethnicity was first harmonized across datasets. The original race field was collapsed into five categories: WHITE, BLACK, HISPANIC, ASIAN, and OTHER. Records containing WHITE were classified as WHITE; records containing BLACK or AFRICAN were classified as BLACK; records containing HISPANIC or LATINO were classified as HISPANIC; records containing ASIAN were classified as ASIAN; and all remaining or missing records were classified as OTHER.

Machine learning preprocessing was implemented using ColumnTransformer. Numeric variables were imputed using K-nearest neighbors imputation with five neighbors and then standardized using StandardScaler. Categorical and binary variables were imputed using the mode and one-hot encoded using OneHotEncoder. OneHotEncoder was configured with drop = first, so the first category of each categorical variable was used as the reference category. Binary variables mainly included variables beginning with hx_, med_, and has_.

All imputation, standardization, and one-hot encoding steps were embedded within the model training pipeline. During cross-validation, the preprocessor was fitted only on the training folds and then applied to the validation fold to avoid information leakage. SMOTE was used only within the training folds to address class imbalance and was not applied to validation folds or exploratory external assessment sets. In the revised analysis, however, the primary model was defined as the race-free, no-imbalance-correction LASSO logistic regression model, and SMOTE was not used in the primary model because it improved discrimination but worsened probability calibration.

### LASSO Feature Selection in the SLE Cohort

LASSO feature selection was first performed in the MIMIC SLE-AKI cohort. Only patients with is_sle = 1 were included. The feature matrix excluded non-predictive variables, including patient identifiers, admission identifiers, admission time, ICU admission time, AKI flag, mortality flag, SLE flag, trajectory labels, and the binary outcome. The remaining variables were entered into the LASSO model as candidate predictors.

The LASSO model was implemented as logistic regression with an L1 penalty, using the liblinear solver and a maximum of 2000 iterations. The penalty strength parameter C was evaluated over 30 candidate values on a logarithmic scale from 0.01 to 10. Hyperparameter tuning was performed using repeated 5-fold stratified cross-validation with five repeats, and ROC AUC was used as the evaluation metric. The model with the highest mean AUC was selected, and variables with non-zero coefficients were exported as core candidate features.

### Core Predictors and Multimodel Risk-Stratification Framework

In the original analysis, the model comparison used six core variables, including race/ethnicity. In the revised analysis, the primary model was changed to a race-free, no-imbalance-correction LASSO logistic regression model including five variables: cr_slope_48h, med_vasopressors, hx_hypertension, med_hcq, and med_vanco. The previous six-variable race-including model was retained only as a sensitivity analysis.

In the revised analysis, the primary model was defined as the race-free, no-imbalance-correction LASSO logistic regression model. This model included five predictors: cr_slope_48h, med_vasopressors, hx_hypertension, med_hcq, and med_vanco. Race/ethnicity was excluded from the primary model. SMOTE was not used in the primary model because it improved discrimination but worsened probability calibration. The previous SMOTE-based six-variable model including race/ethnicity was retained only as a secondary sensitivity analysis.

For the revised primary comparison, the four modeling approaches were evaluated using the five race-free core variables without class imbalance correction. The previous six-variable SMOTE-based comparisons were retained as sensitivity analyses. Four models were compared: standard logistic regression, LASSO logistic regression, random forest, and XGBoost. Standard logistic regression used the liblinear solver. LASSO used L1-penalized logistic regression. The random forest model included 100 trees, a maximum depth of 5, and a minimum leaf size of 4. XGBoost used 100 trees, a learning rate of 0.05, maximum depth of 3, minimum child weight of 3, subsample of 0.8, colsample_bytree of 0.8, L2 regularization coefficient of 1.0, and logloss as the evaluation metric.

### Internal Cross-Validation and Model Performance Evaluation

In the MIMIC cohort, model performance was evaluated using repeated stratified cross-validation. The SLE and non-SLE cohorts were modeled and validated separately. Each model used 5-fold stratified cross-validation repeated 10 times. In each fold, the training set was used for preprocessor fitting and model training, whereas the validation set was used only for prediction and performance evaluation. For SMOTE-based sensitivity analyses, SMOTE oversampling was applied only within the training folds and was not applied to validation folds or exploratory external assessment sets.

ROC curves and AUCs were calculated for each fold. Mean ROC curves were generated by interpolation over a fixed false-positive rate grid followed by averaging. Mean AUC was calculated as the average AUC across all cross-validation folds. The 95% CI was estimated using the standard error of the fold-level AUCs, namely the mean ±1.96 standard errors.

AUC comparisons between the SLE and non-SLE cohorts in Figure 2 were performed using Welch’s independent-samples t test based on the cross-validation AUC distributions.

### Determination of the MIMIC-Derived Optimal Threshold

To support fixed-threshold classification in exploratory external assessment, the script calculated the optimal Youden index threshold in the MIMIC SLE cohort using the final LASSO SLE model. Specifically, the LASSO pipeline trained on the complete MIMIC SLE cohort was used to predict each patient’s probability of a progressive trajectory. The ROC curve was then calculated, and the threshold maximizing sensitivity minus false-positive rate was selected as the MIMIC-derived cutoff. During exploratory external assessment, the threshold was not reoptimized to avoid overfitting in small assessment datasets.

### Multivariable Logistic Regression and OR Estimation

To evaluate the independent associations between the six core variables of the previous race-including model and the progressive AKI trajectory, a multivariable logistic regression model was fitted in the MIMIC SLE-AKI cohort. This analysis used the same preprocessing workflow as the prediction model, including KNN imputation, standardization, and one-hot encoding, but did not use SMOTE because OR estimation should not be based on artificially oversampled data.

ORs and 95% CIs were primarily calculated using the Logit model in statsmodels. For continuous variables, the OR corresponded to the change in risk associated with a one-standard-deviation increase in the standardized variable. For categorical variables, the OR corresponded to the comparison with the one-hot encoded reference category. If the statsmodels Logit model failed because of complete separation or numerical instability, sklearn logistic regression was used, and 95% CIs for ORs were estimated by bootstrap as a backup procedure.

### Propensity Score Matching Analysis of HCQ Use and AKI Progression

To explore the association between HCQ use and the progressive AKI trajectory, propensity score matching was conducted among patients with SLE-AKI in the MIMIC cohort. The HCQ exposure variable was med_hcq. The propensity score model included age, sex, race/ethnicity, minimum creatinine within 48 hours, history of hypertension, history of chronic kidney disease, infection status, and vasopressor use. The actual variables included depended on their availability in the dataset.

Propensity scores were estimated using logistic regression. Numeric variables were imputed using the median, categorical variables were imputed using the mode, and dummy encoding was applied. Matching was performed using nearest-neighbor matching, in which each HCQ user was matched to one non-HCQ control. The matching caliper was 0.1, and controls were not reused.

Covariate balance before and after matching was assessed using standardized mean differences. After matching, the progressive AKI rate was compared between HCQ users and non-users, and the between-group difference was tested using the chi-square test. The OR for the association between HCQ use and AKI progression was estimated using a logistic regression model containing only med_hcq, and the 95% CI was estimated using 1000 bootstrap resamples.

### Sensitivity Analyses

Two major types of sensitivity analyses were performed. The first assessed the stability of core feature coefficients across different modeling scenarios. L1-penalized logistic regression models were refitted in the original dataset, in the dataset after removing aminoglycoside variables, and in a subgroup excluding patients exposed to aminoglycosides. This analysis was performed separately in the SLE and non-SLE cohorts. Coefficient directions and magnitudes were compared for med_hcq, med_vasopressors, hx_hypertension, med_vanco, cr_slope_48h, and race_WHITE.

The second sensitivity analysis evaluated the incremental risk-ranking contribution of the 48-hour creatinine slope. Model A included only baseline variables, comorbidities, infection status, and medication variables. Model B added cr_slope_48h to Model A. Both models were evaluated separately in the SLE and non-SLE cohorts using repeated 5-fold stratified cross-validation with five repeats. Each patient received multiple out-of-fold predicted probabilities across repeated cross-validation; these probabilities were averaged before AUC calculation. The AUCs of Model A and Model B were compared using the DeLong test.

### Interaction Analysis Between SLE and HCQ

To evaluate whether the HCQ association was specific to the SLE disease context, an interaction model was constructed in the combined SLE and non-SLE cohort. The interaction term was defined as is_sle_x_med_hcq = is_sle × med_hcq. The model included SLE status, HCQ use, the SLE × HCQ interaction term, 48-hour creatinine slope, vasopressor use, hypertension history, vancomycin use, race/ethnicity, age, sex, infection status, chronic kidney disease, coronary artery disease, and heart failure, depending on variable availability in the dataset.

The interaction model was fitted using logistic regression. Continuous variables were imputed using KNN imputation and standardized; categorical variables were imputed using the mode and one-hot encoded. ORs for the HCQ main effect and the SLE × HCQ interaction term were obtained by exponentiating model coefficients, and 95% CIs were estimated using 1000 bootstrap resamples.

### eICU Exploratory External Assessment

Before external assessment, race/ethnicity categories were harmonized, and is_sle was converted to an integer variable. If core predictors were missing, numeric variables were imputed as 0 and race/ethnicity was imputed as OTHER. To improve comparability between SLE and non-SLE patients, propensity score matching was performed in the eICU exploratory assessment analysis. Matching variables included age, sex, race/ethnicity, 48-hour creatinine slope, vasopressor use, hypertension history, HCQ use, vancomycin use, chronic kidney disease, and infection status, depending on availability. Propensity scores were estimated using logistic regression. Nearest-neighbor matching was performed, with each SLE patient matched to up to three non-SLE controls. Controls were not reused. If an SLE patient was matched to at least one control, the patient was included in the matched dataset.

During exploratory external assessment, models were not retrained. Instead, the complete pipelines saved from MIMIC were loaded directly. SLE patients were predicted using the MIMIC SLE model, and non-SLE patients were predicted using the MIMIC non-SLE model. The primary performance metric was AUC, and 95% CIs were calculated using 2000 bootstrap resamples. AUC differences between SLE and non-SLE patients were estimated using an independent bootstrap method, with the difference, 95% CI, and two-sided P value reported.

### NWICU Exploratory External Assessment

Propensity score matching between SLE and non-SLE patients was also performed in the NWICU exploratory assessment analysis. The matching covariates used in the current code were 48-hour creatinine slope, vasopressor use, hypertension history, HCQ use, and vancomycin use. Propensity scores were estimated using logistic regression. Although the code output described the matching as 1:1 PSM, the actual implementation searched for up to three nearest non-SLE neighbors for each SLE patient and then removed duplicate controls after merging. Therefore, the more accurate methodological description is nearest-neighbor propensity score matching with up to three controls per SLE patient and removal of duplicate controls.

The NWICU exploratory assessment also used the MIMIC-saved model pipelines directly without retraining. Performance metrics included AUC, bootstrap 95% CI, area under the precision-recall curve (AUPRC), and event rate. AUC differences between the SLE and non-SLE cohorts were compared exploratorily using a normal approximation Z test based on the bootstrap standard deviation.

### Exploratory Assessment in the Local 18-Patient Cohort

In the local assessment cohort, continuous predicted probabilities were used to calculate AUC and Brier score. The primary LASSO model assigned a predicted probability of 0.529 to the only progressive patient. However, two non-progressive patients had predicted probabilities of 0.568, and several additional non-progressive patients had probabilities close to that of the progressive patient. Therefore, continuous-probability ranking was imperfect, yielding an AUC of 0.824. At the MIMIC-derived threshold of 0.3723, the model identified the only progressive patient but also generated false-positive classifications among non-progressive patients. Because the local cohort contained only 18 patients and one progressive event, all local assessment metrics were considered exploratory.

The local exploratory assessment directly loaded the MIMIC-saved SLE model pipelines and evaluated the LASSO, standard logistic regression, random forest, and XGBoost models. ROC and precision-recall curves were plotted, and AUC and AUPRC were calculated. Because the local assessment cohort included only 18 patients and very few events, the local assessment results were considered feasibility and directional evidence rather than definitive evidence of model performance.

Subsequent local risk stratification and confusion matrix analyses focused on the LASSO model. The binary classification threshold preferentially used the MIMIC-derived cutoff rather than reoptimizing the threshold in the local cohort. If the MIMIC-derived cutoff file could not be found, the script calculated a Youden threshold in the local cohort, but that result was considered exploratory only. The confusion matrix reported sensitivity, specificity, positive predictive value, and negative predictive value.

### Nomogram and Web Calculator Parameter Export

The final part of the main analysis script provided a function for exporting model parameters for nomogram construction and interactive web calculator implementation. This function loaded the SLE multivariable logistic regression pipeline used for Figure 3 and extracted the preprocessor, classifier, feature names, standardization parameters, model intercept, and coefficients.

For continuous variables standardized by StandardScaler, coefficients were transformed back to the original variable scale using the training-set means and standard deviations, and the intercept was adjusted accordingly. Coefficients for categorical and binary one-hot encoded variables were retained unchanged. The script also exported the minimum and maximum values of cr_slope_48h in the MIMIC SLE cohort to define the input range for nomogram or web-based implementation.

### Software and Reproducibility

LCMM trajectory modeling was performed in R and mainly relied on the lcmm, readr, dplyr, tidyr, purrr, tibble, and ggplot2 packages. Machine learning modeling and exploratory external assessment were performed in Python and mainly relied on pandas, numpy, scikit-learn, imbalanced-learn, xgboost, statsmodels, scipy, matplotlib, seaborn, and joblib.

Non-overlap sensitivity analysis. To evaluate the influence of structural predictor-outcome overlap, the revised primary race-free LASSO model risk score was applied to predict a post-48-hour hard renal endpoint (creatinine doubling or death within days 2-7 after AKI onset). Patients meeting the endpoint within 48 hours were excluded as baseline prevalent. Discrimination and calibration were assessed using AUC, Brier score, calibration intercept, and calibration slope.

The Python scripts used a random seed of 2026 and set PYTHONHASHSEED = 0. The R script set random seeds according to the number of latent classes when fitting multiclass LCMMs. All models, figures, performance metrics, and intermediate datasets were saved in timestamped output directories to support traceability and reproducibility.

## Supplementary Tables

### Supplementary Table 1. LCMM Model Selection Results in the MIMIC Cohort

| **Number of latent classes** | **Log-likelihood** | **AIC** | **BIC** | **Entropy** | **Minimum class proportion** | **Mean posterior classification probability** | **Convergence** |
| --- | --- | --- | --- | --- | --- | --- | --- |
| 1 | -52244.328 | 104496.656 | 104530.663 | NA | 1.000 | NA | 1 |
| 2 | -42080.961 | 84175.922 | 84235.434 | 0.702 | 0.120 | 0.887 | 1 |
| 3 | -32623.003 | 65266.006 | 65351.023 | 0.810 | 0.069 | 0.889 | 1 |
| 4 | -30412.832 | 60851.663 | 60962.186 | 0.759 | 0.020 | 0.849 | 1 |
| 5 | -28507.256 | 57046.512 | 57182.540 | 0.751 | 0.018 | 0.824 | 1 |

Note: LCMM, latent class mixed model; AIC, Akaike information criterion; BIC, Bayesian information criterion. Although the four-class and five-class models had lower BIC values, their minimum class proportions were only 2.0% and 1.8%, respectively, indicating very small classes. The three-class model balanced entropy, mean posterior probability, class proportion, and clinical interpretability and was therefore selected as the primary trajectory model.

### Supplementary Table 2. Posterior Probabilities and Slope-Based Mapping of the Three LCMM Classes in MIMIC

| **LCMM class** | **n** | **Mean posterior assignment probability** | **First time window** | **Last time window** | **Mean creatinine in first time window** | **Mean creatinine in last time window** | **Absolute change** | **Relative change** | **Clinical label** |
| --- | --- | --- | --- | --- | --- | --- | --- | --- | --- |
| 1 | 3075 | 0.868 | 1 | 7 | 4.151 | 1.335 | -2.817 | -0.679 | Recovery |
| 2 | 2518 | 0.867 | 1 | 7 | 2.040 | 3.391 | 1.351 | 0.662 | Progressive |
| 3 | 30785 | 0.933 | 1 | 7 | 1.380 | 1.301 | -0.080 | -0.058 | Stable |

Note: Class 2 showed an increase in creatinine from 2.040 mg/dL to 3.391 mg/dL, with an absolute change of 1.351 mg/dL and a relative change of 66.2%; it was therefore labeled progressive. Class 1 showed a marked decrease in creatinine and was labeled recovery. Class 3 showed a creatinine change close to zero and was labeled stable.

### Supplementary Table 3. LCMM Model Selection Results in the eICU Cohort

| **Number of latent classes** | **Log-likelihood** | **AIC** | **BIC** | **Entropy** | **Minimum class proportion** | **Mean posterior classification probability** | **Convergence** |
| --- | --- | --- | --- | --- | --- | --- | --- |
| 1 | -78996.747 | 158001.494 | 158037.227 | NA | 1.000 | NA | 1 |
| 2 | -65332.499 | 130678.998 | 130741.531 | 0.718 | 0.116 | 0.886 | 1 |
| 3 | -53402.379 | 106824.758 | 106914.092 | 0.809 | 0.055 | 0.884 | 1 |
| 4 | -49620.011 | 99266.022 | 99382.155 | 0.712 | 0.041 | 0.823 | 1 |
| 5 | -46394.677 | 92821.354 | 92964.288 | 0.754 | 0.015 | 0.822 | 1 |

Note: The three-class eICU model had an entropy of 0.809, a minimum class proportion of 5.5%, and a mean posterior classification probability of 0.884, supporting the use of independent three-class LCMM labels for exploratory external assessment in eICU.

### Supplementary Table 4. Complete Exploratory External Assessment Performance Metrics in eICU

| **Model** | **Cohort** | **N** | **Events** | **Event rate** | **AUC** | **AUC 95% CI** | **P value** |
| --- | --- | --- | --- | --- | --- | --- | --- |
| LASSO | SLE | 94 | 6 | 0.064 | 0.797 | 0.608–0.946 | 0.306 |
| LASSO | Non-SLE | 282 | 22 | 0.078 | 0.680 | 0.530–0.817 | 0.306 |
| Logistic Regression | SLE | 94 | 6 | 0.064 | 0.725 | 0.548–0.894 | 0.691 |
| Logistic Regression | Non-SLE | 282 | 22 | 0.078 | 0.679 | 0.522–0.814 | 0.691 |
| Random Forest | SLE | 94 | 6 | 0.064 | 0.745 | 0.488–0.943 | 0.782 |
| Random Forest | Non-SLE | 282 | 22 | 0.078 | 0.706 | 0.568–0.842 | 0.782 |
| XGBoost | SLE | 94 | 6 | 0.064 | 0.734 | 0.531–0.902 | 0.894 |
| XGBoost | Non-SLE | 282 | 22 | 0.078 | 0.718 | 0.576–0.841 | 0.894 |

Note: This table is based on independent LCMM labels in eICU after propensity score matching. Differences in AUC between the SLE and non-SLE cohorts did not reach statistical significance for any model.

### Supplementary Table 5. Complete Exploratory External Assessment Performance Metrics in NWICU

| **Model** | **Cohort** | **N** | **Events** | **Event rate** | **AUC** | **AUC 95% CI** | **AUPRC** | **PR baseline** |
| --- | --- | --- | --- | --- | --- | --- | --- | --- |
| LASSO | SLE | 49 | 3 | 0.061 | 0.964 | 0.891–1.000 | 0.667 | 0.061 |
| LASSO | Non-SLE | 140 | 6 | 0.043 | 0.831 | 0.634–0.973 | 0.261 | 0.043 |
| Logistic Regression | SLE | 49 | 3 | 0.061 | 0.964 | 0.881–1.000 | 0.698 | 0.061 |
| Logistic Regression | Non-SLE | 140 | 6 | 0.043 | 0.821 | 0.623–0.973 | 0.257 | 0.043 |
| Random Forest | SLE | 49 | 3 | 0.061 | 0.848 | 0.563–1.000 | 0.434 | 0.061 |
| Random Forest | Non-SLE | 140 | 6 | 0.043 | 0.762 | 0.481–0.971 | 0.216 | 0.043 |
| XGBoost | SLE | 49 | 3 | 0.061 | 0.928 | 0.813–1.000 | 0.433 | 0.061 |
| XGBoost | Non-SLE | 140 | 6 | 0.043 | 0.790 | 0.533–0.978 | 0.342 | 0.043 |

Note: The NWICU SLE cohort included only three events; therefore, AUC and AUPRC estimates may be unstable and should be interpreted as exploratory external assessment results. AUPRC, area under the precision-recall curve; PR baseline equals the event rate.

### Supplementary Table 6. Bootstrap Optimism Correction for the Revised Primary Race-Free No-Imbalance-Correction LASSO Model in the MIMIC SLE Cohort

| Metric | Apparent | Optimism | Optimism-Corrected |
| --- | --- | --- | --- |
| AUC | 0.761 | 0.055 | 0.706 |
| Brier Score | 0.086 | -0.006 | 0.092 |
| Calibration Intercept | 0.086 | 0.140 | -0.054 |
| Calibration Slope | 1.353 | 0.406 | 0.947 |

Note: Apparent performance was calculated after fitting the final LASSO pipeline on the full MIMIC SLE cohort and therefore differs slightly from the repeated cross-validation AUC reported in the main text. Bootstrap optimism correction was performed using 1000 resamples. For each bootstrap sample, the revised primary modeling pipeline, including preprocessing and LASSO logistic regression without class imbalance correction, was refitted. The previous SMOTE-based pipeline was retained only as a sensitivity analysis. Because the number of progressive events was limited, optimism-corrected calibration intercepts and slopes should be interpreted descriptively.

### Supplementary Table 7. Calibration Metrics of the Revised Primary Race-Free LASSO Model Across All Cohorts

| cohort | n | events | AUC | Brier | CalInt | CalSlope |
| --- | --- | --- | --- | --- | --- | --- |
| MIMIC-SLE | 279 | 26 | 0.761 | 0.086 | 0.086 | 1.353 |
| eICU | 1129 | 151 | 0.551 | 0.737 | -1.038 | 0.022 |
| NWICU | 49 | 5 | 0.659 | 0.083 | -0.342 | 0.984 |
| Local | 18 | 1 | 0.824 | 0.124 | -0.815 | 0.876 |

Note: Calibration metrics were evaluated using the revised primary race-free, no-imbalance-correction LASSO model. Outcome labels differed by cohort: MIMIC used derivation-cohort LCMM labels; eICU calibration used independent eICU LCMM labels; NWICU used MIMIC-aligned rule-based labels based on relative creatinine change within 7 days; and the local cohort used MIMIC-aligned rule-based labels based on 0-, 24-, and 48-hour creatinine values. Therefore, calibration and AUC estimates across cohorts should not be directly compared, pooled, or ranked. Brier score, calibration intercept, and calibration slope were computed for each cohort. A non-overlap sensitivity analysis assessed the primary model risk score against a post-48-hour hard renal endpoint (creatinine doubling or death), with substantially attenuated discrimination (AUC 0.593). Estimates from NWICU (n=49, 5 events) and the local cohort (n=18, 1 event) should be interpreted as exploratory due to very small event counts.

### Supplementary Table 8. SMOTE-Based Sensitivity Comparison Between the Previous Six-Variable Race-Including Model and the Five-Variable Race-Free Model

| Model | AUC | Brier Score | Calibration Intercept | Calibration Slope |
| --- | --- | --- | --- | --- |
| 6-variable (with race) | 0.87 | 0.179 | -1.139 | 1.355 |
| 5-variable (race-free) | 0.845 | 0.192 | -1.142 | 1.323 |

Note: This comparison used the same preprocessing and LASSO logistic regression pipeline (including SMOTE) to isolate the effect of race/ethnicity removal. The revised primary model, however, is the race-free, no-imbalance-correction model (without SMOTE), reported in Supplementary Tables 6 and 7. Removal of race/ethnicity resulted in only a modest decrease in AUC and similar calibration metrics, supporting the selection of the race-free model as the revised primary model.

### Supplementary Table 9. Comparison of Class Imbalance Handling Strategies in the MIMIC SLE Cohort

| Strategy | AUC | Brier Score | Calibration Intercept | Calibration Slope |
| --- | --- | --- | --- | --- |
| No Correction | 0.761 | 0.086 | 0.086 | 1.353 |
| SMOTE | 0.845 | 0.192 | -1.142 | 1.323 |
| Cost-Sensitive | 0.836 | 0.220 | -1.206 | 1.697 |

Note: All models were fitted using the race-free candidate predictor set. The no-imbalance-correction model was selected as the primary model because it demonstrated superior calibration (Brier score closest to zero, calibration intercept nearest zero, calibration slope nearest 1). SMOTE improved apparent discrimination but produced less favorable calibration metrics, consistent with the phenomenon that synthetic oversampling shifts predicted probabilities away from observed event rates. The six-variable SMOTE-based model (including race/ethnicity) reported in the original analysis is retained as a sensitivity analysis. The no-correction row corresponds to the apparent performance of the revised primary race-free, no-imbalance-correction model. Bootstrap optimism-corrected metrics for this model are reported separately in Supplementary Table 6.

## Supplementary Figures

**Supplementary Figure 1. AKI trajectories in the MIMIC, eICU, and NWICU cohorts.**
The figure shows the mean serum creatinine trajectories and 95% CI bands for the stable, progressive, and recovery AKI trajectories across the three databases. All three databases demonstrated three clinically interpretable trajectory patterns, although the trajectory starting points, slopes, and magnitudes of change differed, indicating cross-database heterogeneity.

**Supplementary Figure 2. LASSO feature selection in the non-SLE cohort.**
The figure shows LASSO feature selection results in the non-SLE AKI cohort. The 48-hour creatinine slope remained an important predictor, whereas HCQ showed no clear predictive contribution.

**Supplementary Figure 3. Disease-specific interaction analysis of HCQ.**
The forest plot shows the ORs and 95% CIs for the HCQ main effect and the SLE × HCQ interaction term. The HCQ main-effect OR was 0.678 (95% CI, 0.293–1.331), and the SLE × HCQ interaction OR was 0.575 (95% CI, 0.239–1.390), indicating a protective direction that did not reach statistical significance.

**Supplementary Figure 4. ROC comparison for eICU exploratory external assessment.**
The figure shows ROC curves and AUC forest plots for four models in the SLE and non-SLE cohorts after 1:3 propensity score matching in eICU. The LASSO model achieved an AUC of 0.797 in the SLE cohort and 0.680 in the non-SLE cohort.

**Supplementary Figure 5. ROC comparison for NWICU exploratory external assessment.**
The figure shows ROC curves and AUC forest plots for four models in the SLE and non-SLE cohorts after propensity score matching in NWICU. The LASSO model achieved an AUC of 0.964 in the SLE cohort and 0.831 in the non-SLE cohort.

**Supplementary Figure 6. ROC and precision-recall curves in the local assessment cohort.**
The figure shows ROC and precision-recall curves for four models in the local 18-patient assessment cohort. Because the cohort included only one progressive event, AUC and AUPRC estimates are highly unstable and should be considered exploratory. For the primary LASSO model, continuous-probability discrimination was imperfect, with an AUC of 0.824. Therefore, local assessment should be interpreted as feasibility and directional evidence rather than definitive evidence of model performance.

**Supplementary Figure 7. Individual risk stratification in the local assessment cohort.
The figure shows LASSO-predicted probabilities for 18 patients in the local assessment cohort. The only progressive patient had a predicted probability of 0.529. Two non-progressive patients had predicted probabilities of 0.568, which were higher than that of the progressive patient, and several additional non-progressive patients had probabilities close to the progressive case. These findings indicate partial but imperfect risk separation. Because the local cohort included only one progressive event, individual risk stratification results should be interpreted as exploratory.**

**Supplementary Figure 8. Confusion matrix in the local assessment cohort.
The MIMIC-derived threshold of 0.3723 was applied for binary classification in the local assessment cohort. At this threshold, the model identified the single progressive patient but also classified five non-progressive patients as progressive, yielding sensitivity of 1.000, specificity of 0.706, positive predictive value of 0.167, negative predictive value of 1.000, and accuracy of 0.722. Because the cohort included only 18 patients and one progressive event, these threshold-based metrics are exploratory and should not be considered robust estimates of clinical classification performance.**

### Supplementary Figure 9. Calibration assessment of the revised primary race-free LASSO model across cohorts. Calibration plots compare predicted probabilities with observed event proportions for the primary LASSO model in the MIMIC SLE cohort, eICU SLE assessment cohort, NWICU SLE assessment cohort, and local assessment cohort. eICU calibration was evaluated in the full SLE assessment cohort using independent LCMM labels rather than in the PSM subset because the matched SLE subset contained only 6 progressive events. The eICU model probabilities showed severe upward shift when the MIMIC-trained model was applied without recalibration; the median predicted probability was 0.999. Therefore, the eICU Brier score is reported descriptively and should not be interpreted as clinically valid probability calibration. NWICU and local calibration estimates are exploratory because of very few progressive events.

### Supplementary Figure 10. SMOTE-Based Calibration Comparison Between the Previous Six-Variable Race-Including Model and the Five-Variable Race-Free Sensitivity Model

Calibration plots comparing the original six-variable model (A) and the five-variable race-free model (B). Both models used the SMOTE-based pipeline. This figure compares SMOTE-based sensitivity models and does not represent the revised primary no-imbalance-correction model. Both models show similar calibration patterns, with the race-free model retaining comparable discrimination (AUC=0.845 vs. 0.870) and calibration (Brier score=0.192 vs. 0.179) to the original model.
